# Empirical design of a variant quality control pipeline for whole genome sequencing data using replicate discordance

**DOI:** 10.1038/s41598-019-52614-7

**Published:** 2019-11-06

**Authors:** Robert P. Adelson, Alan E. Renton, Wentian Li, Nir Barzilai, Gil Atzmon, Alison M. Goate, Peter Davies, Yun Freudenberg-Hua

**Affiliations:** 1 Litwin-Zucker Center for Alzheimer’s Disease, The Feinstein Institute for Medical Research, Northwell Health, Manhasset, New York, 11030 USA; 2 Ronald M. Loeb Center for Alzheimer’s Disease and Department of Neuroscience, Icahn School of Medicine at Mount Sinai, New York, New York, 10029 USA; 3 Robert S. Boas Center for Genomics & Human Genetics, The Feinstein Institute for Medical Research, Northwell Health, Manhasset, New York, 11030 USA; 4 Institute for Aging Research, Albert Einstein College of Medicine, Bronx, New York, 10461 USA; 5 Faculty of Natural Sciences, University of Haifa, Haifa, 31905 Israel; 6 Ronald M. Loeb Center for Alzheimer’s Disease and Departments of Neuroscience, Genetics and Genomic Sciences, and Neurology, Icahn School of Medicine at Mount Sinai, New York, New York, 10029 USA; 7 Division of Geriatric Psychiatry, Zucker Hillside Hospital, Northwell Health, Glen Oaks, New York, 11004 USA

**Keywords:** Genetic variation, Next-generation sequencing

## Abstract

The success of next-generation sequencing depends on the accuracy of variant calls. Few objective protocols exist for QC following variant calling from whole genome sequencing (WGS) data. After applying QC filtering based on Genome Analysis Tool Kit (GATK) best practices, we used genotype discordance of eight samples that were sequenced twice each to evaluate the proportion of potentially inaccurate variant calls. We designed a QC pipeline involving hard filters to improve replicate genotype concordance, which indicates improved accuracy of genotype calls. Our pipeline analyzes the efficacy of each filtering step. We initially applied this strategy to well-characterized variants from the ClinVar database, and subsequently to the full WGS dataset. The genome-wide biallelic pipeline removed 82.11% of discordant and 14.89% of concordant genotypes, and improved the concordance rate from 98.53% to 99.69%. The variant-level read depth filter most improved the genome-wide biallelic concordance rate. We also adapted this pipeline for triallelic sites, given the increasing proportion of multiallelic sites as sample sizes increase. For triallelic sites containing only SNVs, the concordance rate improved from 97.68% to 99.80%. Our QC pipeline removes many potentially false positive calls that pass in GATK, and may inform future WGS studies prior to variant effect analysis.

## Introduction

Next-generation sequencing (NGS), including whole genome sequencing (WGS) and whole exome sequencing (WES), is increasingly applied in clinical diagnostics and treatment development as the demand for precision medicine expands to more conditions and therefore more patients. There are a variety of error sources from sample collection through analysis, including sample contamination, the use of multiple operators, mispriming over or excesses of private variation, machine failure, and DNA degradation^1,2^. False positive variant calls may adversely affect genetic analysis by reducing the power to identify potential risk-modifying associations or by introducing spurious findings^3,4^. There are three general ways to validate NGS variant identification—Sanger sequencing, same-sample replicates, and reference samples^5–8^.

To ensure confidence in the NGS data used in research and clinical settings, NGS data require rigorous quality control (QC). Several WES QC pipelines have been described^9,10^, which use the Genome Analysis Tool Kit (GATK) Variant Quality Score Recalibration (VQSR) approach as their backbones while enhancing GATK’s output by utilizing various hard filters to further screen data based on specific QC metrics. However, no objectively evaluated WGS QC pipeline had been developed until very recently^11^, and this pipeline did not utilize duplicate samples in determining QC filter thresholds or to prioritize filters based on efficacy, and it only considered biallelic variants. WGS studies typically use at least one hard filter based on output parameters from variant calling, but the exact filters and threshold values employed are often arbitrary or not empirically determined^12–14^. In previous studies, multiallelic (non-biallelic) variants were systematically removed in QC steps prior to downstream analysis^11,15,16^, as they were broadly deemed low in quality. However, as sample sizes in sequencing studies increase^10,17,18^, the prevalence of multiallelic variants rises^19^. There may be functional multiallelic variants, and their removal would impact the results of functional analysis of variants. Therefore, high-quality multiallelic variants need to be taken into account in order to calculate meaningful risk burdens and genetic associations, and for analysis pipelines and procedures to have the capacity to robustly scale up for very large datasets.

Here we designed a post-GATK WGS QC pipeline that uses replicate genotype discordance to optimize QC metrics derived from GATK best practices and VQSR, in a dataset-specific manner. Replicate genotype discordance, rather than Sanger sequencing or using reference samples, was chosen as the validation method because of its ease of genome-wide application. Furthermore, replicate genotypes were used in determining high-confidence benchmark genotypes by the Genome in a Bottle Consortium^20^. Our pipeline, which includes variant-level, genotype-level, and sample-level filters, quantifies the efficacy of each filtering step.

## Results

### Empirical thresholds

**Figure 1:**
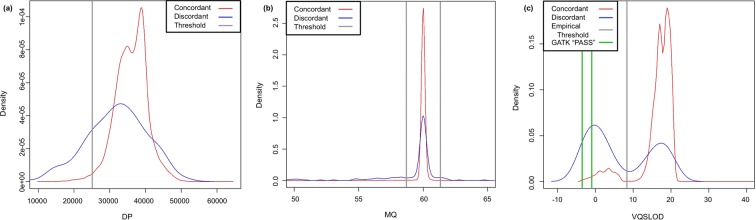
Density plots used to empirically determine thresholds for (A) DP, (B) MQ, and (C) VQSLOD (for SNVs only). These plots compare the densities for discordant and concordant sites, and the thresholds are set in order to maximize the ratio of discordant to concordant sites filtered out. Sites were removed if their total DP was less than 25,000, MQ was less than 58.75 or greater than 61.25, or VQSLOD was less than 7.81 (for SNVs only). The minimum VQSLOD value to be designated “PASS” in GATK was –3.769 for SNVs and –0.961 for indels.

The three empirical variant-level QC thresholds—variant quality score log-odds (VQSLOD), mapping quality (MQ), and overall read depth (DP)—were derived from plots comparing the density curves of each parameter for discordant versus concordant ClinVar-indexed sites (Fig. 1). The VQSLOD for a given variant is a calibrated quality score estimated through the GATK VQSR process that attempts to balance sensitivity and specificity, through a machine learning approach^21^. These dataset-specific thresholds balanced maximization of the ratio of discordant to concordant genotypes removed at each step, as shown in Eq. (1), with removing a high percentage of all discordant genotypes (Supplementary Fig. S1). Thus, remaining sites were removed if VQSLOD was less than 7.81 (for SNVs only), total DP was less than 25,000, or MQ was less than 58.75 or greater than 61.25. These variant-level thresholds were used in all three pipelines.

### QC of ClinVar-indexed variants

We first applied QC to ClinVar-indexed biallelic sites, because ClinVar variants have been extensively investigated by the genetics research community and expert panels^22,23^, and are more likely to be true positives. Statistics of variants retained after sequentially and independently applying each variant-level, genotype-level, and sample-level filter to ClinVar-indexed biallelic sites were gathered (Table 1) and to ClinVar-indexed triallelic sites (Supplementary Table S1). Applying each filter on its own, in addition to sequentially, allowed for each filter’s efficacy to be determined independently.

**Table 1:** Outcome from the hard filters utilized in the QC pipeline, at the variant, genotype, and sample levels, for ClinVar-indexed biallelic sites only.

Variant Level	Site Removal Criterion	Sequential Filtering	Independent Filtering
		# Pass (% Pass), Variants	
—	Monomorphic	38,402 (100)	38,402 (100)
1	Missingness ≥ 5%	38,359 (99.89)	38,776 (99.79)
2	Blacklisted region or LCR	38,359 (100)	38,402 (100)
3	DP < 25,000	37,771 (98.47)	38,098 (98.05)
4	MQ < 58.75 or MQ > 61.25	37,025 (98.02)	37,696 (97.01)
5	VQSLOD < 7.81	36,415 (98.35)	37,080 (95.43)
6	InbreedingCoeff < –0.8	35,751 (98.18)	38,102 (98.06)
**Genotype Level**	**Genotype Removal Criterion**	**# Pass (% Pass)**, **Genotypes**	
7	DP < 10	9,253,660 (99.94)	10,037,482 (99.74)
8	GQ < 20	8,722,641 (94.26)	9,435,150 (93.75)
**Sample Level**	**Sample Removal Criterion**	**# Pass (% Pass)**, **Samples**	
9	Missingness ≥ 10%	259 (100)	259 (100)

The third column represents the number and percentage of variants, genotypes, and samples remaining following the serial application of all nine filters. The fourth column presents the outcome of applying each individual filter to the full ClinVar-indexed dataset (38,402 biallelic variants), indicating each filter’s absolute removal rate. Of 17,585,919 biallelic sites genome-wide, 38,402 matched to ClinVar (which contains 416,908 variants in the 2019-01-02 version used here). Matching was performed using ClinVar version 2019-01-02.

Genotypes were concordant if the non-reference genotypes at a particular variant site were identical among replicate samples. Before QC, 99.38% of the 9,946,118 genotypes at ClinVar-indexed biallelic sites (Table 2) and 89.79% of the 197,876 genotypes at ClinVar-indexed triallelic sites (Supplementary Table S2) were concordant. Our QC steps improved the replicate concordance rate for biallelic variants in the ClinVar subset to 99.73% (Table 2)−99.80% for SNVs and 98.40% for indels. We demonstrated the effect of each filtering step on variant removal, both as independent filters on the full dataset as well as when serially applied. The six sequential variant-level filters removed 7.99% of 38,857 ClinVar-indexed variants (Supplementary Data S1), including 74.87% of 386 variants with at least one discordant genotype (Supplementary Data S2) and the two genotype-level filters removed 5.80% of the 9,259,509 remaining genotypes. The sample-level missingness filter did not remove any samples—missingness ranged from 5.63% to 7.19%. When applied independently, filtering on VQSLOD removed the most variant sites (4.57%), while at the genotype level the GQ filter removed the most genotypes (6.25%).

**Table 2:** Non-reference concordance rates after running each variant-level filter in the QC pipeline in succession, for ClinVar-indexed biallelic sites only.

Variant Filter	Site Removal Criterion	Concordance Rate of Passing Sites (%)	Change in Rate (%)
—	Monomorphic	99.375	—
1	Missingness ≥ 5%	99.473	+0.098
2	Within blacklisted region or LCR	99.473	0
3	DP < 25,000	99.563	+0.090
4	MQ < 58.75 or MQ > 61.25	99.695	+0.132
5	VQSLOD < 7.81	99.725	+0.030
6	InbreedingCoeff < –0.8	99.729	+0.004

These values were calculated following removal of non-‘PASS’ sites according to GATK HaplotypeCaller. A pair of genotypes is concordant when the genotypes of a duplicate pair are identical. The concordance change was always positive or zero. Prior to QC, 99.375% of the 9,946,118 replicate genotypes at ClinVar-indexed biallelic sites were concordant. Following QC, 99.729% of the 8,722,641 remaining genotypes were concordant. Matching was performed using ClinVar version 2019-01-02.

In order to gauge the efficacy of each variant-level filter at removing likely false-positive sites, concordance rate at each step was calculated under sequential conditions (Table 2) and independent conditions (Table 3). A filter was ranked higher if its removal rate of discordant genotypes relative to concordant genotypes was greater, calculated using Eq. (1). For ClinVar-indexed biallelic sites, the variant missingness filter was more than 5 times more efficient at removing discordant genotypes than the next best filter, MQ. Throughout the sequential QC process, the concordance rate increased with each QC filter step (Table 2), an indication that applying any of these variant hard filters improves the dataset quality after using GATK, as measured using concordance as a proxy. For ClinVar-indexed triallelic sites, the concordance rate increased at higher magnitude with each QC step, but reached a lower final concordance rate of 94.22% (Supplementary Table S2). Although 7.32% of concordant genotypes were removed in QC, 74.87% of discordant genotypes were removed at ClinVar-indexed biallelic sites. The VQSLOD filter, which is commonly used in practice, accounted for removal of 55.96% of discordant genotypes when applied to all ClinVar-indexed biallelic sites (only 12.16% of all genotypes removed by the VQSLOD filter, however, were discordant).

**Table 3:** Ranking of variant-level filters for ClinVar-indexed biallelic sites, and genome-wide biallelic and triallelic sites.

Rank	Filter	Negative Predictive Value			Specificity		
		Discordances among Discarded Genotypes (%)			% of Discordant Genotypes Removed		
		ClinVar Biallelic	All Biallelic	All Triallelic	ClinVar Biallelic	All Biallelic	All Triallelic
1	Missingness	87.65	1.98	42.55	18.39	0.03	34.92
2	MQ	16.19	8.85	42.91	48.70	55.38	79.98
3	DP	13.97	20.72	45.97	27.46	19.21	53.34
4	VQSLOD*	12.16	6.77	41.15	55.96	68.65	99.03
5	InbreedingCoeff	2.25	2.31	29.62	4.40	3.65	37.76

The filters are ranked in order from greatest to lowest preference for filtering out discordant genotypes. Negative predictive value refers to a filter’s ability to remove discordant genotypes (true negatives) and minimize the number of concordant genotypes removed (false negatives). Specificity refers to a filter’s ability to identify and remove discordant genotypes (true negatives) and minimize the number of discordant genotypes retained (false positives). Matching was performed using ClinVar version 2019-01-02.

*Filter applied to biallelic and triallelic sites involving only SNVs.

There are assertion criteria for each variant entry in the ClinVar^23^. These assertion criteria indicate the number of submitters (one or multiple), whether or not assertion criteria or evidence were provided in the submissions, and whether the assertion criteria conflict between multiple submitters. The six different assertion criteria for the ClinVar-indexed variants in this study vary from no assertion criteria provided (0 stars) to 3 stars (reviewed by an expert panel, the strongest assertion in our dataset). For ClinVar-indexed biallelic sites, the percentage of total sites removed (*p* = 0.022 by Fisher’s exact test), concordances removed (*p* < 0.0001), and discordances removed (*p* < 0.0001) varied significantly among the different assertion criteria (Supplementary Table S3). Notably, one discordant and three concordant 3-star ClinVar-indexed variants, all located with 400 kilobases on chromosome 2, were removed in QC (Supplementary Table S4).

### QC of genome-wide biallelic and triallelic sites

**Figure 2:**
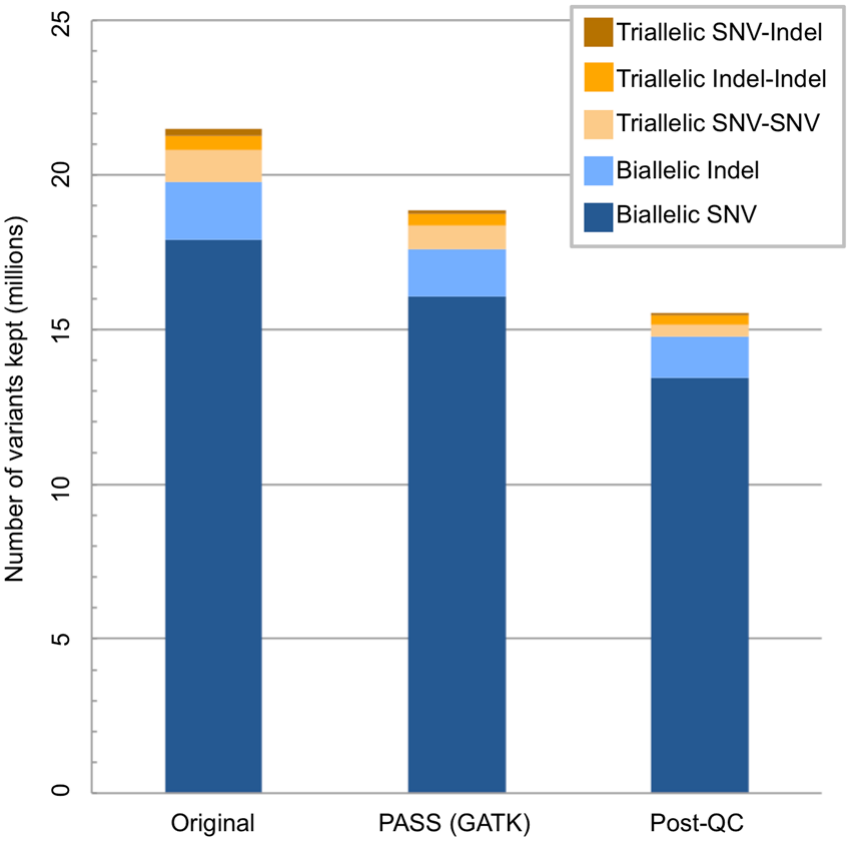
The distribution of biallelic and triallelic sites. This distribution is shown for the original dataset, following removal of non-‘PASS’ variants (according to GATK HaplotypeCaller), and following application of all variant-level filters.

We applied the QC pipeline designed for ClinVar-indexed variants to genome-wide biallelic and triallelic sites. Before QC, 98.53% of 30,137,375 non-reference replicate genotypes at genome-wide biallelic sites (98.69% at SNVs and 96.89% at indels) and 84.16% of 2,604,018 non-reference replicate genotypes at genome-wide triallelic sites were concordant. Variant, genotype, and sample counts of all biallelic and triallelic sites throughout the QC process show the removal rate at each step (Table 4). Our QC steps improved the replicate non-reference concordance rate for genome-wide biallelic variants from 98.53% to 99.69% (Table 5)—from 98.69% to 99.81% for SNVs and from 96.89% to 98.53% for indels. Among genome-wide triallelic sites, the replicate non-reference concordance rate increased from 84.16% to 94.36%. The six sequential variant-level filters removed 16.06% of all biallelic sites and 42.20% of all triallelic sites. Among the 9,260,109 removed non-reference genotypes at biallelic sites, 1,941,431 (20.97%) were discordant. Additionally, 16.45% of biallelic SNVs and 12.03% of biallelic indels were filtered out (Fig. 2). The two genotype-level filters removed 0.54% of the remaining genotypes among biallelic sites and 8.18% of the remaining genotypes among triallelic sites. The sample-level missingness filter in the biallelic pipeline did not remove any samples—missingness ranged from 0.26% to 1.07% following QC. When considering all triallelic sites after QC, sample-level missingness was considerable, ranging from 6.87% to 13.67%. However, when only triallelic sites containing two SNVs were considered, no samples failed QC, with post-QC sample-level missingness ranging from 4.85% to 8.31%. Following QC, triallelic sites containing only SNVs comprised 2.32% of sites (Supplementary Table S5). This fraction is similar to the proportion of multiallelic SNVs identified in Phase 3 of the 1000 Genomes Project, but lower than the 6.4% from the Exome Aggregation Consortium (ExAc) data^19^, likely due to the smaller sample size in our study and the non-linear increase in the proportion of multiallelic sites with sample size.

**Table 4:** Outcome from the hard filters utilized in the QC pipeline, at the variant, genotype, and sample levels, for genome-wide biallelic and triallelic sites.

Variant Level	Site Removal Criterion	Biallelic, Sequential Filtering	Triallelic, Sequential Filtering
		# Pass (% Pass), Variants	
–	Monomorphic	17,585,919 (100)	1,536,657 (100)
1	Missingness ≥ 5%	17,584,990 (99.99)	1,536,085 (99.96)
2	Blacklisted region or LCR	17,584,990 (100)	1,536,085 (100)
3	DP < 25,000	17,346,931 (98.65)	1,345,292 (87.58)
4	MQ < 58.75 or MQ > 61.25	15,971,098 (92.17)	968,987 (72.03)
5	InbreedingCoeff < –0.8	15,661,311 (98.06)	949,810 (98.02)
6	VQSLOD < 7.81	14,760,982 (94.25)	888,194 (93.51)
**Genotype Level**	**Genotype Removal Criterion**	**# Pass (% Pass)**, **Genotypes**	
7	DP < 10	3,819,276,086 (99.96)	202,424,447 (98.89)
8	GQ < 20	3,800,347,137 (99.50)	187,956,031 (92.85)
**Sample Level**	**Sample Removal Criterion**	**# Pass (% Pass)**, **Samples**	
9	Missingness ≥ 10%	259 (100)	193 (74.52)

These values were calculated following removal of non-‘PASS’ sites according to GATK HaplotypeCaller. The third and fourth columns include results when only variants passing the preceding filter move on to the subsequent filter. If only SNV-SNV triallelic sites are considered for the triallelic pipeline, zero samples are removed in the triallelic pipeline (the missingness for all samples remained below 8.5%).

**Table 5:** Non-reference concordance rate after running each hard filter in the QC pipeline in succession at the variant level, for biallelic and triallelic variants.

Variant Filter	Site Removal Criterion	Concordance Rate of Passing Sites (%)			
		All Biallelic	Biallelic SNVs	Biallelic Indels	All Triallelic
—	Monomorphic	98.532	98.690	96.887	84.155
1	Missingness ≥ 5%	98.533	98.690	96.887	84.155
2	Within blacklisted region or LCR	98.533	98.690	96.887	84.155
3	DP < 25,000	98.798	98.904	97.673	87.570
4	MQ < 58.75 or MQ > 61.25	99.401	99.482	98.536	92.704
5	InbreedingCoeff < –0.8	99.404	99.486	98.529	92.671
6	VQSLOD < 7.81	99.694	99.810	98.529	94.358

These values were calculated following removal of non-‘PASS’ sites according to GATK HaplotypeCaller. A pair of genotypes is concordant when the genotypes of a duplicate pair are identical. The change in concordance rate was always positive. Prior to QC, 98.532% of the 30,137,375 replicate non-reference genotypes at genome-wide biallelic sites were concordant; following QC, 99.694% of the 25,180,411 remaining non-reference genotypes were concordant. Prior to QC, 84.155% of the 2,604,018 replicate genotypes at genome-wide triallelic sites were concordant; following QC, 94.358% of the 1,522,106 remaining genotypes were concordant.

As before, to determine the efficacy of each variant-level filter in the biallelic and triallelic pipelines at removing likely lower-quality sites, the non-reference concordance rate at each step was calculated under sequential conditions (Table 5) and as a stand-alone independent filter (Table 3). In the biallelic pipeline, the variant-level DP filter was more than twice as efficient at removing discordant genotypes (while retaining concordant sites) than the next best filter (MQ), while the variant-level missingness filter removed very few discordant sites. In the triallelic pipeline, most variant-level filters achieved a discordant to concordant genotype removal ratio of approximately 2-to-3. Throughout the sequential QC process, the concordance rate generally increased with each step (Table 5). Among biallelic sites, the six variant-level hard filters removed 14.89% of concordant genotypes and 82.11% of discordant genotypes. The VQSLOD filter accounted for removal of 68.65% of all discordant genotypes when applied on its own. Among triallelic sites, 34.46% of concordant genotypes and 79.19% of discordant genotypes were removed; the VQSLOD filter removed 66.82% of discordant genotypes when applied independently.

The transition/transversion (Ti/Tv) ratio was calculated at each step in the biallelic and triallelic pipelines (Table 6) as a broad quality check for sequencing and SNV quality, as is common practice^9,24–27^. The biallelic sites originally had a Ti/Tv ratio of 2.04, which increased nearly constantly as variant-level filters were applied, reaching a final value of approximately 2.16—similar to the Ti/Tv ratio expected for known variants from reported WGS data^28^. This indicates that, among biallelic sites in this QC process, transversions were removed at a higher rate than transitions. The triallelic SNV-SNV sites (with two SNV alternate alleles) originally had a Ti/Tv ratio of 0.94, which generally decreased as variant-level filters were applied until the final ratio of 0.85 was reached. This change was opposite that of the biallelic Ti/Tv ratio—sites containing a transition were removed at a significantly higher rate than sites containing two transversions in the triallelic pipeline (56.63% and 51.21%, respectively; *p* < 0.0001 by Fisher’s exact test). As indicated in a density plot of VQSLOD (Supplementary Fig. S2), differentiating between transitions and transversions, a greater proportion of transversions than transitions had VQSLOD values below the threshold of 7.81.

**Table 6:** Ti/Tv ratio at each variant-level step in the genome-wide biallelic and triallelic pipelines.

Filter/Step	Biallelic Sites		Triallelic Sites	
	Ti/Tv	Change (%)	Ti/Tv	Change (%)
(1) Original	1.88322	—	1.88322	—
(2) Biallelic (or Triallelic) Only	2.04350	+8.511	0.94341	−0.940
(3) ‘PASS’	2.14108	+4.775	0.96112	+0.018
(4) Missingness	2.14111	+0.001	0.96122	+0.0001
(5) DP	2.14874	+0.356	0.94256	−0.019
(6) MQ	2.14418	−0.212	0.85855	−0.084
(7) InbreedingCoeff	2.14707	+0.135	0.87857	+0.020
(8) SNV VQSLOD	2.16381	+0.780	0.85444	−0.024

Ti/Tv increases by 0.12 (5.9%) among biallelic SNVs, from before GATK is run (step 2) through the end of QC. Ti/Tv decreases by 0.089 (9.4%) among triallelic SNV-containing sites (SNV-SNV and SNV-indel).

Further analysis was conducted to compare the removal rate and discordance rate of rare (MAF ≤ 1%) versus common (MAF ≥ 5%) variants (Supplementary Table S6). The percentage of concordances removed (false negative rate) was not significantly different between rare and common variants (*p* = 0.57). The percentage of discordances removed (true negative rate) was significantly higher for rare variants than for common variants (*p* < 0.0001).

### Triallelic pipeline particulars

The outputs of the triallelic pipeline were also measured by distinguishing between four types of triallelic sites—SNV-SNV (two SNV alternate alleles), SNV-indel (one SNV alternate allele and one indel alternate allele), indel-indel (two indel alternate alleles), and other-indel (one indel alternate allele and one symbolic alternate allele such as ‘*’, which indicates a spanning deletion)^29^. Variant counts of these four types of triallelic sites throughout the QC process show the removal rate at each step (Supplementary Table S7). The six sequential variant-level filters removed 49.92% of SNV-SNV (41.92% without the VQSLOD filter), 32.92% of SNV-indel, 21.88% of indel-indel, and 52.68% of other-indel sites. The concordance rate at each step was calculated under sequential conditions (Supplementary Table S8) and with independent application of the filters. Among triallelic sites, the VQSLOD filter was only applied to SNV-SNV sites; this filter was the most effective of the variant-level filters at removing discordant SNV-SNV genotypes. The DP filter was most effective at removing discordant SNV-indel, indel-indel, and other-indel genotypes while retaining concordant genotypes, and comparable to the VQSLOD filter when eliminating poor SNV-SNV genotypes (Supplementary Table S9).

For all four triallelic subtypes, the concordance rate almost always increased with the sequential application of variant-level filters, as was the case for triallelic sites overall (Supplementary Table S8). At triallelic sites, without applying the VQSLOD filter, the sequential QC process removed 93.40% of SNV-SNV, 62.57% of SNV-indel, 69.49% of indel-indel, and 72.25% of other-indel discordant non-reference genotypes. Due to the use of the VQSLOD filter, a higher percentage of SNV-SNV sites were removed compared to SNV-indel and indel-indel sites (all removed using the same filter thresholds, besides VQSLOD). Our QC pipeline was more effective at removing SNV-SNV discordant genotypes than such genotypes at SNV-indel and indel-indel sites. The removal rate of transition- and transversion-containing triallelic sites is shown in the supplementary data (Supplementary Table S10). The decrease in the Ti/Tv ratio of triallelic sites with sequential application of the variant-level filters was driven by removal of transition-indel sites, while the Ti/Tv ratio of SNV-SNV sites increased.

## Discussion

Our study found that large numbers of variants that passed GATK VQSR contained substantial numbers of discordant genotypes in our cohort (Supplementary Fig. S3). This implies that NGS studies performed on different sequencing platforms may introduce errors that could affect association studies. Many of our findings—the importance of considering triallelic SNV-SNV sites, the benefits of applying hard filters to a GATK variant callset, and the utility of a small number of replicate samples in quality control—can be applied generally to WGS datasets. While the three empirical filter thresholds are dataset-specific, VQSLOD can be filtered on without replicate sites by removing the lower peak in its bimodal distribution. The filters for inbreeding coefficient, variant- and sample-level missingness, GQ, and genotype-level DP can be used across many datasets. Given that the false negative rate did not significantly differ between rare and common variants, this pipeline can be utilized for populations including various ethnicities (given that variant allele frequencies may differ between ethnicities).

The QC pipeline that we implemented worked similarly well for biallelic SNVs and indels (final non-reference concordance rates of 99.81% and 98.53%, respectively) and very well for triallelic SNV-SNV sites (final non-reference concordance rate of 99.80%), but was less successful for other triallelic sites (final non-reference concordance rates ranging from 84.78% to 97.29%). The six hard variant-level filters used in our pipeline removed nearly 75% of discordant genotypes at ClinVar-indexed biallelic sites and more than 82% of discordant genotypes at genome-wide biallelic sites. The genome-wide biallelic pipeline had a specificity (for removing discordances) of 82.11% and sensitivity (for retaining concordances) of 85.11%, while the genome-wide triallelic pipeline had a specificity of 79.19% and sensitivity of 65.54%. However, the removal of triallelic SNV-SNV sites had a specificity of 93.40% and sensitivity of 76.84%. The performance of genome-wide biallelic sites was certainly stronger than that of triallelic sites as a whole, but the triallelic SNV-SNV subset was comparable in performance to genome-wide biallelic sites.

As larger numbers of samples are required for rare variant association studies, investigators often merge NGS data from many different sources in a meta-analysis. If an NGS case/control association study runs all cases on one platform and controls on another, spurious findings could result. Therefore it is preferable for all samples in a WGS dataset to be sequenced using the same platform to minimize the potential introduction of multifactorial errors prior to alignment. Some of these discordances may be due to differences in the sequencing process and machines—the 98.53% concordance rate in this case was lower than the average 99.49% in a previous study that in part looked at concordance among replicate sample genotypes sequenced on identical machines^28^. The use of a small number of duplicate samples (where feasible) is a useful method for identifying variant calls of low confidence in NGS and for determination of empirical thresholds for parameters such as VQSLOD, MQ, and overall DP. However, filtering thresholds derived from GATK’s recommendations or a literature consensus can be used even without the running of some samples in duplicate. Additionally, these QC filters are flexible—stringent or relaxed thresholds can be used depending on general knowledge of a dataset’s quality and the goals of a sequencing project, and any set of these filters can be used to improve different aspects of data quality. For example, if read depth and potential excess heterozygosity are of foremost concern, then filtering on DP and inbreeding coefficient would be useful in improving concordance and callset quality.

As expected, the VQSLOD filter removed the highest percentage of all discordant genotypes of all variant-level filters (55.96% for ClinVar-indexed and 68.65% for genome-wide biallelic sites). This is partly due to the GATK machine learning algorithm ranking all variants using the VQSLOD score, which accounts for multiple sequencing parameters depending on the user’s input into the software. Our empirically derived VQSLOD score cutoff maximized the ratio of removal of biallelic discordant genotypes to concordant genotypes, but it still removed 14.89% the initial 30,137,375 concordant non-reference replicate biallelic genotypes. The generally lower confidence of discordant genotype calls is evidenced by the lower VQSLOD scores indicated in a density plot of sites containing only concordant genotypes versus sites containing one or more discordances (Fig. 1c). Notably, the VQSLOD density plot indicated that this parameter had a bimodal distribution both for concordant and discordant sites, with a majority of discordant sites in the lower-scored peak and a majority of concordant sites in the higher-scored peak. This bimodality was consistent with findings from a previous study^21^. Even without replicate sequencing, a filter which removes variants whose VQSLOD scores fall in the lower peak would remove a high percentage of potential false positives. Furthermore, the VQSLOD filter that we applied to our dataset showed particularly high specificity (99.03%) but low negative predictive value (41.15%) for discordant genotypes when removing triallelic SNV-SNV variants.

An additional interesting finding is the steady increase in Ti/Tv ratio for biallelic sites in all samples with the sequential application of the six variant-level filters. This indicates that a higher percentage of transversions are removed than are transitions, a result of the distribution of VQSLOD scores for transversions being slightly left-shifted (lower) compared to the distribution for transitions. The assignment of lower VQSLOD scores to transversions may originate from transversions being less prevalent than transitions in protein-coding regions of the human genome^30,31^, as well as the lower allele frequency of coding transversions^32^, which is also true in the training set (Ti/Tv = 2.00) utilized in VQSR^28^. Since transversions are less common, when the algorithm comes upon a real transversion in the test set it is less likely to have strong quality parameters and therefore more likely to be assigned a lower VQSLOD score.

Triallelic variants are understudied—with the few focused studies utilizing WES rather than WGS—and therefore studies typically investigate only biallelic variants (for which most sequence analysis tools are constructed)^33^. The results of the present study confirm that triallelic sites as a group are indeed lower in quality than biallelic sites. However, as indicated in our study, many triallelic sites—in particular, SNV-SNV sites—are high in quality and are thus retained through a rigorous QC process identical to that applied to biallelic variants. As would be expected given the higher removal rate of biallelic indels compared to biallelic SNVs, SNV-SNV sites were more concordant throughout QC than sites containing at least one indel variant. It is vital to consider likely true triallelic sites, because there are several well-established Mendelian disease variants that appeared at triallelic sites. One triallelic site harbors the ΔF508 mutation in *CFTR* (rs113993960), causal for cystic fibrosis in the homozygous state, an allele that is present in approximately 1% of Europeans and Americans^34,35^. In the 259-subject dataset used here, three individuals (1.16%) were heterozygous for this mutation, with no homozygotes observed. Additionally, the *MDR1* (*ABCB1*) triallelic SNV *G2677/T/A* is well studied and relevant in inflammatory bowel disease^36^. Both of these ClinVar-indexed sites passed all QC steps in this study. Exclusion of all triallelic sites, a common practice in NGS studies such as the NHLBI Exome Sequencing Project (ESP)^19,37^, would have removed these well-known disease-causing variants from further consideration. In searching for disease-causing alleles in individuals (for diagnosis) and cohorts, especially with increasingly large NGS datasets, it is certainly important to consider including high-quality triallelic sites^19^.

Consistent with our expectation, ClinVar-indexed variants had greater sequencing quality compared to the genome-wide biallelic callset—6.90% of ClinVar-indexed biallelic sites were removed, while 16.06% of genome-wide biallelic sites were filtered out through QC. This genome-wide removal rate is similar to the filtering result in the ADSP WGS pipeline^11^. Additionally, ClinVar-indexed biallelic had a post-GATK concordance rate of 99.38%, while genome-wide biallelic sites had a post-GATK concordance rate of 98.53%. Our findings attest to the value of curated variant databases such as ClinVar, as many variants present in ClinVar have been quality-checked during clinical investigations. Despite this substantial difference in removal rate, both sets of variants had discordant sites as evidenced by the use of duplicate concordance testing. Our genome-wide biallelic pipeline resulted in a similar final concordance rate as the ClinVar-indexed biallelic pipeline (99.73% and 99.69%, respectively), which is evidence for the genome-wide efficacy of this QC methodology.

Although there are many potential follow up studies to this investigation, three particular avenues are of great interest. First, any sequencing study needs to factor in certain sequencing errors, as illustrated in our analysis of discordant genotypes in replicate samples (1.47% of genotypes at biallelic sites and 15.85% of genotypes at triallelic sites were discordant post-GATK), and must determine the source of these errors in order to improve data quality for clinical or research interpretation. Potential sources of these discordances include operator error during sequencing, machine-borne error, differences in sequencing accuracy between machines, DNA degradation or contamination over time between sequencing runs, and differences in library prep kits and chemistry. The list of removed ClinVar-indexed variants is included as a supplementary file. Second, post-GATK analysis of both the original unfiltered data and the filtered data following QC will help determine whether such fine-tuning of hard filters improves the investigation of pathogenic variant burden and other clinically relevant features of interest. Third, the bias of GATK against transversions when assigning VQSLOD scores is interesting, and future investigation may shed more light on this observation. The findings here suggest that using callset-specific hard filters in QC can successfully remove discordant and other lower-quality sites, which is vital for the success of next-generation sequencing analysis.

In summary, we have designed a scalable dataset-specific QC pipeline applicable to GATK variant callsets, as well as other toolkits outputting similar QC parameters. By using replicate samples sequenced on different machines from the same manufacturer, we highlighted the discordant genotypes developed from the use of dissimilar instruments and we utilized these discordances as a proxy for quantifying removal of likely false-positive variants. Triallelic sites were thoroughly investigated, and those sites involving only SNVs were found to be close in quality to biallelic sites. This QC pipeline can be utilized and adapted for many NGS studies of various diseases and control samples, providing a set of higher-quality variant calls and genotypes prior to ensuing analyses.

## Methods

### Ethics approval and consent to participate

This study analyzed de-identified datasets and is not considered human subjects research according to the institutional review board (IRB) at Northwell Health. DNA samples for sequencing were collected with written informed consent in accordance with a protocol approved by the IRB at Montefiore Medical Center and the Committee on Clinical Investigation at the Albert Einstein College of Medicine, Northwell Health, and the National Institute on Aging Genetics Initiative for Late-Onset Alzheimer Disease/National Cell Repository for Alzheimer Disease (NIA-LOAD/NCRAD). This work was carried out in accordance with relevant institutional and governmental guidelines and regulations.

### Whole genome sequencing, alignment, variant genotype calling, and variant annotation

WGS was performed to an average depth of 30 $\times$ in 262 individuals, using purified DNA from peripheral whole blood. The details of subject enrollment were previously described^38^, and principal component analysis showed that 255 subjects are of Ashkenazi Jewish ancestry and seven subjects have European ancestry.

The first batch of 125 subjects were sequenced via WGS by Illumina, Inc., in 2012 using the Illumina HiSeq. 2500, at 30 $\times$ average coverage^39^. A second batch, consisting of 137 new subjects and eight subjects from the first batch (to serve as a subset for QC), was sequenced via WGS by New York Genome Center (NYGC) in 2016 using the Illumina HiSeq X Ten at 30 $\times$ average coverage^28,39^. Library preparation, sequencing protocols, alignment specifications, genotype calling, and primary annotation procedures are provided (Supplementary Text S1; Supplementary Table S11). The WGS parameters differed slightly by sequencing center, while all subsequent alignment and calling parameters were identical (Supplementary Table S11). NYGC performed alignment against the GRCh37 human reference build using the Burrows-Wheeler Aligner (BWA) and variant calling using GATK to generate 25 single-chromosome files in the Variant Call Format (VCF)—one per autosome and sex chromosome, and one for mitochondrial DNA^40^. The quantities of biallelic and multiallelic (triallelic and ≥4 allele) sites were determined before proceeding, and again following the full QC (Supplementary Table S5).

Prior to application of the variant-level filters, samples were removed if they fell under any of three criteria: (1) If it was a duplicate sample sequenced in an earlier batch; (2) if subjects were related with identity by descent (IBD) parameter PI-HAT ≥ 0.3, all members but one from that sibling group were removed; and (3) if a sample had an evidenced sequencing error. Three samples were removed due to first-degree kinship with subjects in the remainder of the cohort. Duplicate samples were used for concordance testing. Samples that were sequenced once were only used when calculating aggregate parameters, such as VQSLOD.

### ClinVar-indexing, biallelic, and triallelic pipelines

**Figure 3:**
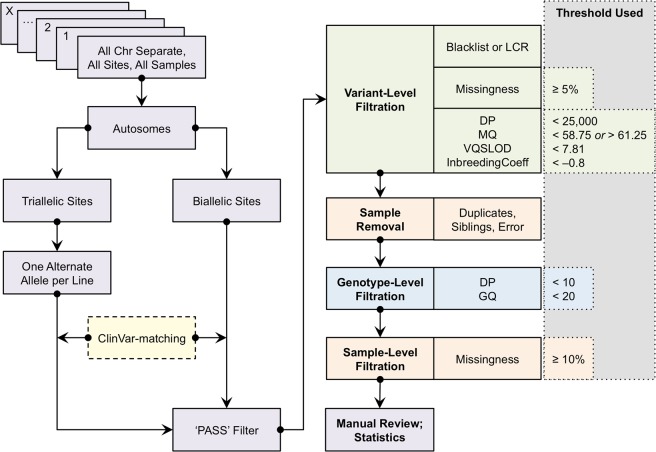
Schematic for the genome-wide biallelic, triallelic, and ClinVar-indexing pipelines. The pipelines include: indexing sites in the full VCF files to the ClinVar database (in the ClinVar-indexing pipeline only), several applications of pre-QC filters and annotations, variant-level filtration, sample-level filtration, genotype-level filtration, a recommended manual review of the final output, and study-specific statistical and association analyses.

We created three distinct pipelines composed of overlapping components, referred to hereafter as the ClinVar-indexing, biallelic, and triallelic pipelines (Fig. 3).

For the ClinVar-indexing pipeline, monomorphic sites were removed. The remaining autosomal variants were checked for matches in the ClinVar database, version 2019-01-02^22,23^. Biallelic sites were read from the original variant callset, while triallelic sites were first split to yield two variant rows each (one per alternate allele). Each variant was disambiguated with a unique identifier of the format “chrom.pos.ref.alt” (CPRA), a concatenation of the chromosome number, GRCh37 position, reference allele, and alternate allele for the variant. Unmatched variants were removed from further consideration in this pipeline. Subsequently, only variants with a “PASS” in the FILTER column of the VCF were considered^11^, which in this instance included single nucleotide variants (SNVs) with a VQSLOD value ≥ –3.769 (Tranche 99.8%) and indels with a VQSLOD value ≥ –0.961 (Tranche 99.0%). The individual ClinVar-indexed autosome VCFs were combined into a single file, and annotated with dbSNP reference SNP ID numbers (rsIDs) from their corresponding ClinVar entries^41^. Two further annotations were added—INDEL and SNV—to indicate the variant type. This annotated file was fed into the filtration portion of the pipeline. Since ClinVar indexing reduces the number of variants substantially, the ClinVar-indexing pipeline was scripted in R using the packages seqMINER, VariantAnnotation, and Biostrings (Supplementary Text S2)^42–44^.

The biallelic pipeline was written in a series of shell scripts (Supplementary Text S3), using bcftools and vcftools, which are adaptable for workflow environments such as Snakemake^40,45,46^. Each autosome VCF was handled individually, rather than concatenating the files as in the ClinVar-indexing pipeline. Again, each variant was disambiguated with a CPRA identifier, and monomorphic and multiallelic sites were removed, and sites with a value besides “PASS” in the FILTER column were removed^10,11,40^.

The triallelic pipeline differed from the biallelic pipeline by the removal of non-triallelic sites and splitting of triallelic sites into one line per allele^47^.

### Filters and threshold determination

A total of nine QC filters were applied in each pipeline (Table 7). These QC filters were applied identically in all three pipelines, with six variant-level filters, two genotype-level filters, and one sample-level filter.

**Table 7:** Hard filters utilized in the QC pipeline, at the variant, genotype, and sample levels.

Variant Level	Site Removal Criterion
1	Missingness ≥ 5%
2	Within blacklisted region or LCR
3	DP < 25,000
4	MQ < 58.75 or MQ > 61.25
5	VQSLOD < 7.81
6	InbreedingCoeff < –0.8
Genotype Level	Genotype Removal Criterion
7	DP < 10
8	GQ < 20
Sample Level	Sample Removal Criterion
9	Missingness ≥ 10%

The thresholds for steps 4 through 6 (DP, MQ, and VQSLOD) were determined empirically, by comparing density plots for those parameters in concordant and discordant variants.

The six variant-level filters can be applied in any order. Each variant position was checked against the UCSC Blacklist and a list of low-complexity regions (LCRs)^48–50^, both of which refer to sequence regions that are difficult to map. Variants overlapping these regions were removed. Variant sites with missingness greater than or equal to 5% were removed^51^; this stringent filter was selected due to our relatively small sample size, whereas more lenient variant missingness filters, such as 10% or 20%, are often used in studies of many thousands of individuals^11,52^. Three additional thresholds—variant-level DP, MQ, and VQSLOD—were empirically determined. These three hard filter thresholds were chosen to balance removing as many discordant genotypes as possible with maximizing the ratio of concordant to discordant genotypes retained, with the ratio based on the formula

1 $$\frac{{\bf{1}}-{\boldsymbol{p}}}{{\boldsymbol{p}}}$$

where *p* is the fraction of genotypes that are discordant and (1 – *p*) is the fraction of genotypes that are concordant. Additional sites were removed if the inbreeding coefficient was less than –0.8, in order to remove sites with excess heterozygosity, as recommended by GATK^10,24,27^.

Genotype-level filters were then applied. A genotype was removed if its read depth (DP) was less than 10, a value used in several previous investigations using WGS at 30 $\times$ coverage^10,11^. Additionally, a genotype was removed if its genotype quality (GQ) was less than 20, because if GQ < 20 there is a > 1% likelihood of the genotype call being false^9^.

Following the removal of low-quality genotypes, missingness was calculated for each sample based on the remaining genotypes. A sample was removed if its missingness was greater than or equal to 10%, a conservative threshold^11,51^.

## Supplementary information

Supplementary Information

Supplementary Data

## Data Availability

All whole genome sequencing data reported in this article will be deposited to the National Institute on Aging Genetics of Alzheimer’s Disease Data Storage Site (NIAGADS). We are currently performing further analyses on this data. In the meantime, reasonable requests for deidentified genomic data should be sent to the corresponding author.
