# Supplementary material for: Empirical design of a variant quality control pipeline for whole genome sequencing data using replicate discordance
Source: Sci Rep. 2019 Nov 6;9:16156. doi: 10.1038/s41598-019-52614-7 (PMC6834861; doi:10.1038/s41598-019-52614-7)
Supplement: Supplementary file 1 — Supplementary Information [file 41598_2019_52614_MOESM1_ESM.pdf]

Supplementary Information for:

Empirical design of a variant quality control pipeline for whole genome sequencing data using replicate discordance

Robert P. Adelson[1], Alan E. Renton[2], Wentian Li[3], Nir Barzilai[4], Gil Atzmon[4,5], Alison M. Goate[6], Peter Davies[1], Yun Freudenberg-Hua[1,7,*]

Affiliations:
[1] Litwin-Zucker Center for Alzheimer's Disease, The Feinstein Institute for Medical Research, Northwell Health, Manhasset, New York 11030, USA
[2] Ronald M. Loeb Center for Alzheimer's Disease and Department of Neuroscience, Icahn School of Medicine at Mount Sinai, New York, New York 10029, USA
[3] Robert S. Boas Center for Genomics & Human Genetics, The Feinstein Institute for Medical Research, Northwell Health, Manhasset, New York 11030, USA
[4] Institute for Aging Research, Albert Einstein College of Medicine, Bronx, New York 10461, USA
[5] Faculty of Natural Sciences, University of Haifa, Haifa 31905, Israel
[6] Ronald M. Loeb Center for Alzheimer's Disease and Departments of Neuroscience, Genetics and Genomic Sciences, and Neurology, Icahn School of Medicine at Mount Sinai, New York, New York 10029, USA
[7] Division of Geriatric Psychiatry, Zucker Hillside Hospital, Northwell Health, Glen Oaks, New York 11004, USA

* Correspondence: Yun Freudenberg-Hua, MD
yfreuden@northwell.edu

Contents **Page**

Contents	Page
Supplementary Figure S1	2
Supplementary Table S1	3
Supplementary Table S2	4
Supplementary Table S3	5
Supplementary Table S4	6
Supplementary Table S5	7
Supplementary Figure S2	8
Supplementary Table S6	9
Supplementary Table S7	10
Supplementary Table S8	11
Supplementary Table S9	12
Supplementary Table S10	13
Supplementary Figure S3	14
Supplementary Text S1	15
Supplementary Table S11	16
Supplementary Text S2	17
Supplementary Text S3	18

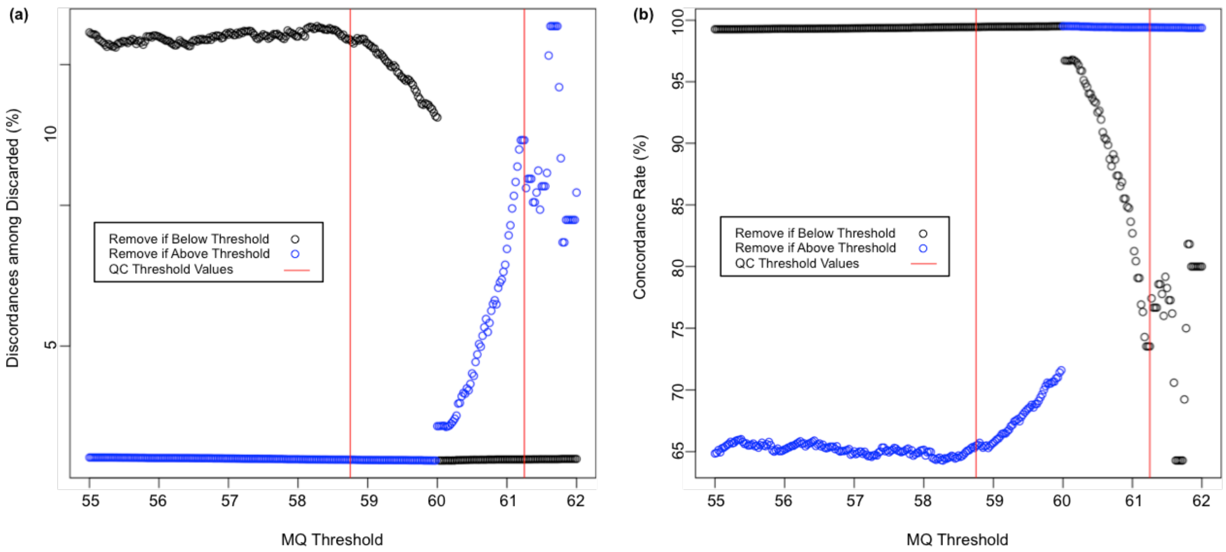

Supplementary Figure S1. Example of empirical determination of a hard filter threshold, for MQ. (A) Various MQ thresholds are plotted versus the discordance rate of variants discarded at each threshold, for ClinVar-indexed variants. The lower removal threshold of MQ $\leq$ 58.75 was chosen after plotting in the aforementioned manner when variants *below* a given MQ threshold (black circles) are removed. The upper removal threshold of MQ $\geq$ 61.25 was chosen after plotting in the aforementioned manner when variants *above* a given MQ threshold (blue circles) are removed. (B) Various MQ thresholds are again plotted, now versus the concordance rate following independent application of each MQ threshold on the unfiltered ClinVar-indexed dataset.

		Sequential Filtering	Independent Filtering
**Variant Level**	**Site Removal Criterion**	**# Pass (% Pass), Variants**	
–	Monomorphic	764 (100)	764 (100)
1	Missingness ≥ 5%	749 (96.86)	783 (96.67)
2	Blacklisted region or LCR	749 (100)	810 (100)
3	DP < 25,000	710 (95.95)	757 (93.46)
4	MQ < 58.75 or MQ > 61.25	674 (94.93)	751 (92.72)
5	VQSLOD < 7.81	612 (90.80)	693 (85.56)
6	InbreedingCoeff < –0.8	554 (90.52)	737 (90.99)
**Genotype Level**	**Genotype Removal Criterion**	**# Pass (% Pass), Genotypes**	
7	DP < 10	141,659 (98.73)	205,377 (97.90)
8	GQ < 20	94,733 (66.87)	136,717 (65.17)
**Sample Level**	**Sample Removal Criterion**	**# Pass (% Pass), Samples**	
9	Missingness ≥ 10%	0 (0)	259 (100)

Supplementary Table S1. **Outcome from the hard filters utilized in the QC pipeline, at the variant, genotype, and sample levels, for ClinVar-indexed triallelic sites only** These values were calculated following removal of non-'PASS' sites according to GATK HaplotypeCaller. The third column represents the number and percentage of variants, genotypes, and samples remaining following the serial application of all nine filters. The fourth column presents the outcome of applying each individual filter to the full ClinVar-indexed dataset (810 triallelic sites), indicating each filter's absolute removal rate.

Variant Filter	Site Removal Criterion	Concordance Rate of Passing Sites (%)	Change in Rate (%)
–	Monomorphic	89.791	–
1	Missingness ≥ 5%	91.622	+1.831
2	Within blacklisted region or LCR	91.622	0
3	DP < 25,000	92.254	+0.632
4	MQ < 58.75 or MQ > 61.25	93.769	+1.515
5	VQSLOD < 7.81	94.281	+0.512
6	InbreedingCoeff < –0.8	94.224	-0.057

Supplementary Table S2. **Non-reference concordance rates after running each variant-level filter in the QC pipeline in succession, for ClinVar-indexed triallelic sites only**. These values were calculated following removal of non-'PASS' sites according to GATK HaplotypeCaller. A pair of genotypes is concordant when the genotypes of a duplicate pair are identical. The change in concordance rate was generally positive. Prior to QC, 89.791% of the 197,876 replicate genotypes at ClinVar-indexed biallelic sites were concordant. Following QC, 94.224% of the 94,733 remaining genotypes were concordant.

Variant Filter	Site Removal Criterion	Concordance Rate of Passing Sites (%)						
		3 stars	2 stars	1 star		0 stars		
		Expert	Multiple	Conflict	Single	No Interp	No Assert	All Biallelic
–	Monomorphic	98.913	99.546	99.661	99.350	100.000	98.987	99.375
1	Missingness ≥ 5%	98.913	99.619	99.661	99.455	100.000	99.167	99.473
2	Blacklisted or LCR	98.913	99.619	99.661	99.455	100.000	99.167	99.473
3	DP < 25,000	100.000	99.673	99.706	99.553	100.000	99.265	99.563
4	MQ < 58.75 or > 61.25	100.000	99.742	99.850	99.688	100.000	99.510	99.695
5	InbreedingCoeff < –0.8	100.000	99.754	99.849	99.730	100.000	99.501	99.725
6	VQSLOD < 7.81	100.000	99.765	99.847	99.733	100.000	99.493	99.729
**# Non-Monomorphic Sites**		92	6,827	2,066	26,615	37	2,765	38,402
**Total Sites Removed (%)**		4.348	6.430	5.276	7.133	8.108	7.342	6.903
**Concordances Removed (%)**		3.297	6.224	5.100	6.754	8.108	6.869	6.572
**Discordances Removed (%)**		100.000	51.613	57.143	61.850	—	53.571	59.583

Supplementary Table S3. Non-reference concordance rates after running each variant-level filter in the QC pipeline in succession, for ClinVar-indexed biallelic sites only, with ClinVar assertion criteria for variant pathogenicity provided. These values were calculated following removal of non-'PASS' sites according to GATK HaplotypeCaller. A pair of genotypes is concordant when the genotypes of a duplicate pair are identical. The number of stars corresponds to the strength of the assertion criteria, and is defined by ClinVar. The percentage of concordances removed is the false negative rate. Matching was performed using ClinVar version 2019-01-02. "Expert" = reviewed by expert panel. "Multiple" = criteria provided, multiple submitters, no conflicts. "Conflict" = criteria provided, conflicted interpretations. "Single" = criteria provided, single submitter. "No Interp" = no interpretation for the single variant. "No Assert" = no assertion criteria provided.

rsID	Chrom	Pos (GRCh37)	Ref	Alt	Pathogenicity	Variation ID	Gene	Discordant?
rs587779109	2	47702054	T	TAGG	Likely benign	90772	MSH2	No
rs55927047	2	48010654	C	G	No known pathogenicity	89295	MSH6	Yes
rs56320267	2	48032326	T	C	No known pathogenicity	89411	MSH6	No
rs189436849	2	48032717	T	A	Likely benign	89416	MSH6	No

Supplementary Table S4. List of 3-star ClinVar-indexed biallelic sites removed after running all variant-level filters in the QC pipeline in succession. These three sites have the highest assertion criteria (3 stars, reviewed by an expert review panel) among the ClinVar-indexed variants in the 262-sample dataset. Variant position, reference allele, and alternate allele are from human reference build GRCh37. rsID = reference SNP cluster ID. Chrom = chromosome. Pos = position in GRCh37. Ref = reference allele in GRCh37. Alt = alternate non-reference allele in GRCh37. Variation ID = variant ID in the ClinVar database. Discordant? = whether or not one or more of the eight sequencing replicate genotype pairs were discordant at this site.

	Post-GATK			Post-QC		
	Total (% of All)	Variant Type per Chromosome		Total (% of All)	Variant Type per Chromosome	
		Min (%)	Max (%)		Min (%)	Max (%)
**Biallelic Sites**	19,782,177 (86.02)	82.84	87.19	14,760,982 (88.79)	85.25	89.80
SNV	17,911,386 (77.89)	74.17	79.41	13,414,514 (80.69)	76.54	82.23
Indel	1,870,791 (8.14)	7.41	8.67	1,346,468 (8.10)	7.03	8.70
**Triallelic Sites**	2,149,532 (9.35)	8.68	11.50	888,194 (5.34)	4.58	5.91
SNV-SNV	1,031,687 (4.49)	4.11	5.51	385,795 (2.32)	2.19	2.73
SNV-Indel	216,296 (0.94)	0.82	1.19	88,383 (0.53)	0.49	0.68
Indel-Indel	460,709 (2.00)	1.80	2.57	288,506 (1.74)	1.60	2.18
Other-Indel	440,840 (1.92)	1.66	2.35	125,510 (0.75)	0.67	1.00
**4+ Allele Sites**	1,064,347 (4.63)	4.09	5.66	1,064,347 (6.40)	5.51	8.85

Supplementary Table S5. The number of biallelic and multiallelic sites in the overall dataset. Biallelic sites are reported in total, and subdivided into SNVs and indels. Triallelic sites are reported in total, and subdivided into SNV-SNV, SNV-indel, indel-indel, and other-indel sites. The minimum and maximum percentages of an individual chromosome's variants falling into these categories are also shown. These values are reported for the post-GATK (prior to removal of non-'PASS' sites according to GATK HaplotypeCaller) and post-QC (using the biallelic or triallelic pipeline, as appropriate) call sets for biallelic and triallelic sites. Sites with 4+ alleles were not filtered but are included in calculating post-QC percentages for completeness.

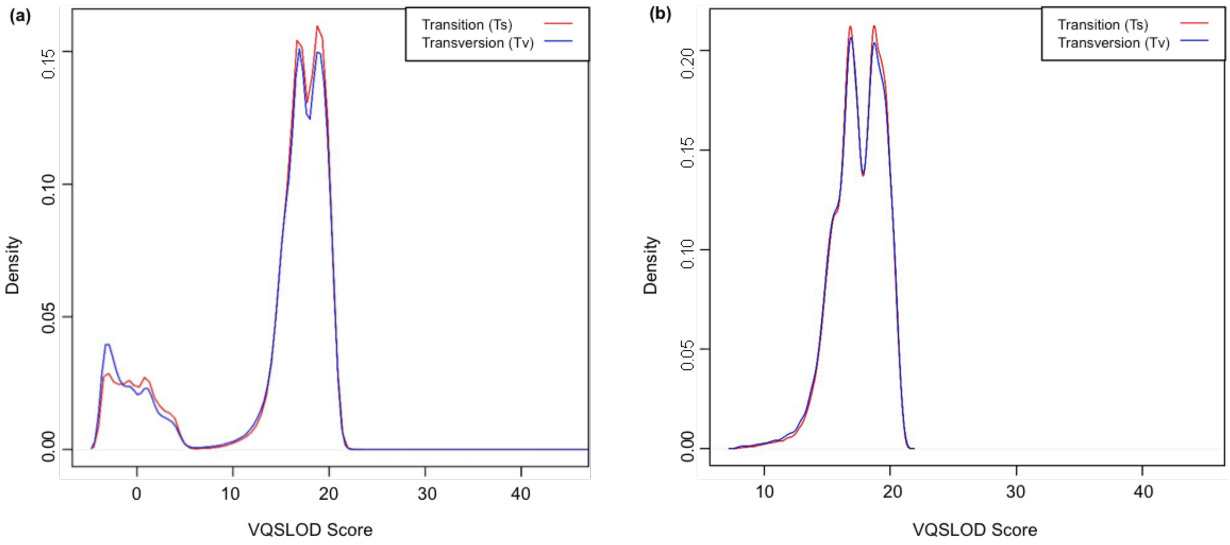

Supplementary Figure S2. Density plots of VQSLOD scores for transitions and transversions in biallelic SNVs. The plots show the VQSLOD distribution (A) before and (B) after all variant-level QC filters were applied**.** All SNVs with VQSLOD scores less than 7.81 were removed from the call set, and some sites with scores greater than 7.81 were removed at other steps.

		Concordance Rate of Passing Sites (%)		
		Rare (MAF ≤ 1%)	Common (MAF ≥ 5%)	All Biallelic
–	Monomorphic	99.995	99.308	99.375
1	Missingness ≥ 5%	99.995	99.308	99.473
2	Blacklisted or LCR	99.995	99.308	99.473
3	DP < 25,000	99.998	99.415	99.563
4	MQ < 58.75 or > 61.25	100.000	99.752	99.695
5	InbreedingCoeff < –0.8	100.000	99.755	99.725
6	VQSLOD < 7.81	100.000	99.830	99.729
**Total Sites Removed (%)**		15.909	14.916	16.064
**Concordances Removed (%)**		14.827	14.872	14.891
**Discordances Removed (%)**		97.778	79.196	82.111

Supplementary Table S6. Non-reference concordance rates after running each variant-level filter in the QC pipeline in succession, for biallelic sites only, comparing rare and common variants. These values were calculated following removal of non-'PASS' sites according to GATK HaplotypeCaller. A pair of genotypes is concordant when the genotypes of a duplicate pair are identical. Rare variants have a minor allele frequency (MAF) ≤ 1%, while common variants have a MAF ≥ 5%. The percentage of concordances removed is the false negative rate.

Variant Level	Site Removal Criterion	# Pass (% Pass)				
		SNV-SNV	SNV-Indel	Indel-Indel	Other-Indel	All Triallelic
–	Monomorphic	770,363 (100)	131,759 (100)	369,300 (100)	265,235 (100)	1,536,657 (100)
1	Missingness ≥ 5%	769,993 (99.95)	131,723 (99.97)	369,234 (99.98)	265,235 (99.96)	1,536,085 (99.96)
2	Blacklisted or LCR	769,993 (100)	131,723 (100)	369,234 (100)	265,135 (100)	1,536,085 (100)
3	DP < 25,000	653,737 (84.90)	119,817 (90.96)	346,156 (93.75)	225,582 (85.08)	1,345,292 (87.58)
4	MQ < 58.75 or > 61.25	459,172 (70.24)	89,866 (75.00)	292,753 (84.57)	127,196 (56.39)	968,987 (72.03)
5	InbreedingCoeff < –0.8	447,411 (97.44)	88,383 (98.35)	288,506 (98.55)	125,510 (98.67)	949,810 (98.02)
6	VQSLOD < 7.81	385,795 (86.23)	88,383 (–)	288,506 (–)	125,510 (–)	888,194 (93.51)

Supplementary Table S7. **Outcome from the variant-level hard filters utilized in the QC pipeline, for triallelic sites**. Triallelic sites are subdivided into SNV-SNV, SNV-indel, indel-indel, and other-indel. These values were calculated following removal of non-'PASS' sites according to GATK HaplotypeCaller. The third through seventh columns include results with serial application of the QC filters. Filter 6 only removed SNV-SNV sites falling below the VQSLOD threshold.

Variant Filter	Site Removal Criterion	Concordance Rate of Passing Sites (%)				
		SNV-SNV	SNV-Indel	Indel-Indel	Other-Indel	All Triallelic
–	Monomorphic	97.682	91.531	92.875	73.251	84.155
1	Missingness ≥ 5%	97.682	91.531	92.875	73.251	84.155
2	Blacklisted or LCR	97.682	91.531	92.875	73.251	84.155
3	DP < 25,000	98.011	93.921	95.040	77.071	87.570
4	MQ < 58.75 or > 61.25	99.367	96.062	97.306	84.856	92.704
5	InbreedingCoeff < –0.8	99.369	96.037	97.288	84.776	92.671
6	VQSLOD < 7.81	99.797	96.037	97.288	84.776	94.358

Supplementary Table S8. Non-reference concordance rate after running each variant-level filter in the QC pipeline in succession, for triallelic sites. Triallelic sites are subdivided into SNV-SNV, SNV-indel, indel-indel, and other-indel. These values were calculated following removal of non-'PASS' sites according to GATK HaplotypeCaller. Filter 6 only removed SNV-SNV sites falling below the VQSLOD threshold. A pair of genotypes is concordant when the genotypes of a duplicate pair are identical. The change in concordance rate was generally positive.

		Negative Predictive Value					Specificity				
		**Discordances among Discarded Genotypes (%)**					**% of Discordant Genotypes Removed**				
**Rank**	**Filter**	**SNV-SNV**	**SNV-Indel**	**Indel-Indel**	**Other-Indel**	**All Triallelic**	**SNV-SNV**	**SNV-Indel**	**Indel-Indel**	**Other-Indel**	**All Triallelic**
1	MQ	33.45	36.40	29.75	45.57	42.91	95.17	73.83	70.96	81.33	79.98
2	VQSLOD*	41.15	—	—	—	41.15	99.03	—	—	—	99.03
3	DP	36.86	40.10	33.80	49.51	45.97	43.45	52.04	48.65	56.16	53.34
4	InbreedingCoeff	30.85	31.25	22.38	28.01	29.62	36.76	33.87	24.54	20.25	37.76
5	Missingness	38.51	35.90	28.51	45.46	42.55	32.25	30.96	21.67	37.30	34.92

Supplementary Table S9. Ranking of variant-level filters for genome-wide triallelic sites. The filters are ranked in order from greatest to lowest preference for filtering out discordant genotypes. Negative predictive value refers to a filter's ability to remove discordant genotypes (true negatives) and minimize the number of concordant genotypes removed (false negatives). Specificity refers to a filter's ability to identify and remove discordant genotypes (true negatives) and minimize the number of discordant genotypes retained (false positives).

*: Filter applied to biallelic and triallelic sites involving only SNVs.

Type of Site	Major Allele	% of Sites Removed in QC
Ts-Tv	Ts	45.6
	Tv	45.8
Tv-Tv	Tv	58.6
Ts-Indel	Ts	59.6
	Indel	46.2
Tv-Indel	Tv	50.4
	Indel	44.3

Supplementary Table S10. **Removal rate of triallelic SNV-SNV and SNV-indel sites in QC**. SNV alleles are subdivided into transitions (Ts) and transversions (Tv), and the type of major allele (Ts, Tv, or indel) is indicated. Ts-Tv sites are removed at a lower rate than Tv-Tv sites, while Tv-indel sites are removed at a lower rate than corresponding Ts-indel sites.

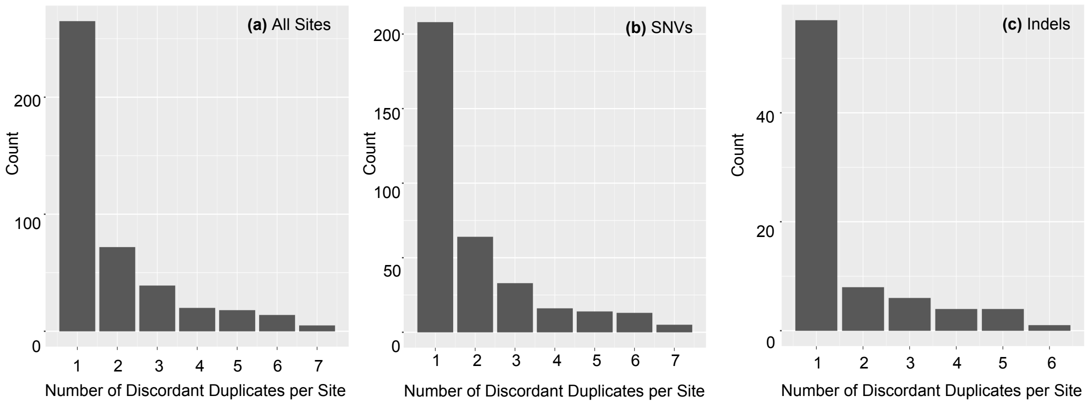

Supplementary Figure S3. Number of ClinVar-indexed discordant duplicates per site. Discordances are shown for (A) all sites (SNVs and indels), (B) SNVs only, and (C) indels only. There were 386 discordant sites in total (306 SNVs, 80 indels).

Supplementary Text S1. Library preparation, whole genome sequencing (WGS), and post-processing protocol from New York Genome Center (NYGC).

Pre-processing

WGS data were processed using the NYGC automated pipeline. Paired-end 150 bp reads were re-aligned to the GRCh37 human reference using the Burrows-Wheeler Aligner (BWA-MEM v0.7.8) and processed using the Genome Analysis Tool Kit (GATK) best-practices workflow that includes marking of duplicate reads by the use of the Picard tools (v1.83), local realignment around indels, and base quality score recalibration (BQSR) using GATK v3.2.0.

Variant Discovery

Variant discovery was a two-step process. All variant discovery used GATK v3.2.0 HaplotypeCaller, which was run on each sample separately in genomic VCF (GVCF) mode. This produced an intermediate GVCF for each sample. GVCFs of multiple samples were then run through a joint genotyping step to produce a multi-sample VCF. Variant filtration was performed using Variant Quality Score Recalibration (VQSR), a machine learning based algorithm used to identify annotation profiles of variants that are likely to be real, and assigns a VQSLOD score to each variant.

Variant Annotation

Variant effects annotation was performed using SnpEff, bcftools, and in-house software. Other functional annotations include variant frequencies in different populations from 1000 Genomes Project (1000GP), ExAC, and dbSNP 138; cross-species conservation scores from phyloP, Genomic Evolutionary Rate Profiling (GERP), and phastCons; functional prediction scores from PolyPhen-2 and SIFT; variant disease annotations from OMIM and ClinVar; regulatory approaches from Regulome; and KEGG pathway annotations.

Structural Variant Analysis

Structural variations (SVs) were called per batch of around 100 samples using GenomeSTRiP. GenomeSTRiP combines discordant read pair analysis with read depth analysis in order to identify large deletions (> 100 bp, < 1 Mb). Joint calling of multiple samples and the use of population scale filters help to distinguish real SVs from artifacts. All deletions annotated as PASS in the GenomeSTRiP results were then processed with in-house scripts. First, deletions were merged within the same sample to remove redundant calls. Next, they were merged across samples and annotated with gene overlap, overlap with known deletions (based on 1000GP and Database of Genomic Variants), and overlap of predicted breakpoints with repeat regions. Using this annotation with repeat regions we filter out SVs whose breakpoints fall into sequences with extensive mapping ambiguity or repeat content and that are likely artifact calls due to mismappings.

Principle Component Analysis

Variant calls generated by the Variant Discovery workflow were used to infer population structure using EIGENSOFT software.

(A)

Feature	Illumina	NYGC
Number of samples	125	145
Amount of input DNA	1 μg	1 μg
Library prep kit	Illumina TruSeq PCR-Free	Illumina TruSeq PCR-Free
Insert size	470 bp ±10%	570 bp ±10%
Sequencing platform	Illumina HiSeq 2500	Illumina HiSeq X

(B)

Program	Version/Build
Reference	GRCh37
Alignment	BWA 0.7.8
Duplicate reads	Picard 1.83
Indel realignment	GATK 3.4-0
Merge	Picard 1.83
GATK indels	GATK 3.4-0
GATK recalibration	GATK 3.4-0

Supplementary Table S11. **Parameters used for whole genome sequencing, alignment, and calling.** (A) Parameters of whole genome sequencing, by sequencing center. (B) Parameters used for whole genome alignment and calling, identical for both sequencing centers, all performed at NYGC.

Supplementary Text S2. Select code for the ClinVar-indexing Pipeline (written in R), indicating key steps in matching variants to the ClinVar database.

```
#Load GRCh37 ClinVar data VCF file, from: ftp.ncbi.nlm.nih.gov/pub/
clinvar <- VCFloci("clinvar.vcf.gz")

#Match autosomal variants to ClinVar data
for (chrNum in 1:22) {
      dataFile <-paste("filename.chr",chrNum,".vcf.gz",sep="")
      clinvarChr <- clinvar[which(clinvar$CHROM == chrNum),]

      #Create a disambiguated variant ID
      clinvarChrAll <-paste(clinvarChr$CHROM,clinvarChr$POS,
      clinvarChr$REF,clinvarChr$ALT,sep=".")

      charZero <- character(0)
      clinvarMatches <- list()
      for (i in 1:NROW(clinvarChr)) {
            posTemp <- clinvarChr$POS[i]
            tabixTemp <- tabix.read(dataFile,paste(chrNum,":",posTemp,
                  "-",posTemp,sep=""))

            #If unmatched, don't add to list; if matched, add to list
            charTest <- identical(tabixTemp,charZero)
            if (charTest == FALSE) {
                  clinvarMatches <- rbind(clinvarMatches,tabixTemp)
            }
      }
}
```

Supplementary Text S3. Biallelic pipeline shell script, used for filtering low-quality biallelic variants from full autosomal variant call files.

```
for i in 1 2 3 4 5 6 7 8 9 10 11 12 13 14 15 16 17 18 19 20 21 22
do

#Remove all but 1 sibling per sibling group and sample errors, and
#keep only biallelic sites; keep duplicates to find concordance rates
vcftools --gzvcf chr${i}.vcf.gz --remove-indv sample1 --remove-indv
sample2 --remove-indv sample3 --remove-indv sample4 --max-alleles 2 --
min-alleles 2 --stdout --recode --recode-INFO-all | bgzip >
chr${i}.biallelic.vcf.gz
bcftools stats chr${i}.biallelic.vcf.gz > chr${i}.biallelic.stats.txt


#Keep sites annotated as "PASS" by GATK
vcftools --gzvcf chr${i}.biallelic.vcf.gz --remove-filtered-all --
stdout --recode --recode-INFO-all | bgzip > chr${i}.pass.vcf.gz
bcftools stats chr${i}.pass.vcf.gz > chr${i}.pass.stats.txt

#Keep sites with variant missingness ≤ 5%
vcftools --gzvcf chr${i}.pass.vcf.gz --max-missing 0.05 --stdout --
recode --recode-INFO-all | bgzip > chr${i}.var_miss.vcf.gz
bcftools stats chr${i}.var_miss.vcf.gz > chr${i}.var_miss.stats.txt

#Keep sites with overall DP > 25,000
vcffilter -f  "DP > 25000" chr${i}.var_miss.vcf.gz |
bgzip > chr${i}.var_dp.vcf.gz
bcftools stats chr${i}.var_dp.vcf.gz > chr${i}.var_dp.stats.txt

#Keep sites with MQ in the range 58.75 to 61.25
vcffilter -f  "MQ > 58.75 & MQ < 61.25" chr${i}.var_dp.vcf.gz |
bgzip > chr${i}.mq.vcf.gz
bcftools stats chr${i}.mq.vcf.gz > chr${i}.mq.stats.txt

#Keep sites with Inbreeding Coefficient > -0.8
vcffilter -f  "InbreedingCoeff > ( 0 - 0.8 )" chr${i}.mq.vcf.gz |
bgzip > chr${i}.inbreed.vcf.gz
bcftools stats chr${i}.inbreed.vcf.gz > chr${i}.inbreed.stats.txt

#Separate out indels, which are not filtered by VQSLOD
vcftools --gzvcf chr${i}.inbreed.vcf.gz --keep-only-indels --stdout --
recode --recode-INFO-all | bgzip > chr${i}.indels.vcf.gz
```

```
#Separate out SNVs, which are then kept if VQSLOD > 7.81
vcftools --gzvcf chr${i}.inbreed.vcf.gz --remove-indels --stdout --
recode --recode-INFO-all | vcffilter -f "VQSLOD > 7.81" |
bgzip > chr${i}.snvs.vcf.gz
bcftools stats chr${i}.snvs.vcf.gz > chr${i}.snvs.stats.txt

#Combine indels and filtered SNVs into one VCF file
bcftools concat chr${i}.indels.vcf.gz chr${i}.snvs.vcf.gz | bcftools
sort | bgzip > chr${i}.combined.vcf.gz
bcftools stats chr${i}.combined.vcf.gz > chr${i}.combined.stats.txt

#Remove 1 sample per duplicate pair
vcftools --gzvcf chr${i}.combined.vcf.gz --remove-indv sample5
--remove-indv sample6 --remove-indv sample7 --remove-indv sample8
--remove-indv sample9 --remove-indv sample10 --remove-indv sample11
--remove-indv sample12 --stdout --recode --recode-INFO-all |
bgzip > chr${i}.no_dupl.vcf.gz
bcftools stats chr${i}.no_dupl.vcf.gz > chr${i}.no_dupl.stats.txt

#Keep genotypes with genotype-level DP > 10
vcffilter -g "DP > 10" chr${i}.no_dupl.vcf.gz |
bgzip > chr${i}.geno_dp.vcf.gz
bcftools stats chr${i}.geno_dp.vcf.gz > chr${i}.geno_dp.stats.txt

#Keep genotypes with GQ > 20
vcffilter -g "GQ > 20" chr${i}.geno_dp.vcf.gz |
bgzip > chr${i}.gq.vcf.gz
bcftools stats chr${i}.gq.vcf.gz > chr${i}.gq.stats.txt

#Calculate sample missingness; remove samples with missingness ≥ 10%
vcftools --gzvcf chr${i}.gq.vcf.gz --missing-indv --out chr${i}.gq
vcftools –gzvcf chr${i}.gq.vcf.gz --remove-indv sample 13 --stdout --
recode --recode-INFO-all
done
```
